# Unmanned Aerial Vehicles (UAVs) for Surveying Marine Fauna: A Dugong Case Study

**DOI:** 10.1371/journal.pone.0079556

**Published:** 2013-11-04

**Authors:** Amanda Hodgson, Natalie Kelly, David Peel

**Affiliations:** 1 Murdoch University Cetacean Research Unit, School of Veterinary and Life Sciences, Murdoch University, Murdoch, Western Australia, Australia; 2 CSIRO Computational Informatics and Wealth from Oceans National Research Flagship, Hobart, Tasmania, Australia; 3 Australian Marine Mammal Centre, Kingston, Tasmania, Australia; University of Western Ontario, Canada

## Abstract

Aerial surveys of marine mammals are routinely conducted to assess and monitor species’ habitat use and population status. In Australia, dugongs (*Dugong dugon*) are regularly surveyed and long-term datasets have formed the basis for defining habitat of high conservation value and risk assessments of human impacts. Unmanned aerial vehicles (UAVs) may facilitate more accurate, human-risk free, and cheaper aerial surveys. We undertook the first Australian UAV survey trial in Shark Bay, western Australia. We conducted seven flights of the *ScanEagle* UAV, mounted with a digital SLR camera payload. During each flight, ten transects covering a 1.3 km^2^ area frequently used by dugongs, were flown at 500, 750 and 1000 ft. Image (photograph) capture was controlled via the Ground Control Station and the capture rate was scheduled to achieve a prescribed 10% overlap between images along transect lines. Images were manually reviewed *post hoc* for animals and scored according to sun glitter, Beaufort Sea state and turbidity. We captured 6243 images, 627 containing dugongs. We also identified whales, dolphins, turtles and a range of other fauna. Of all possible dugong sightings, 95% (CI = 90%, 98%) were subjectively classed as ‘certain’ (unmistakably dugongs). Neither our dugong sighting rate, nor our ability to identify dugongs with certainty, were affected by UAV altitude. Turbidity was the only environmental variable significantly affecting the dugong sighting rate. Our results suggest that UAV systems may not be limited by sea state conditions in the same manner as sightings from manned surveys. The overlap between images proved valuable for detecting animals that were masked by sun glitter in the corners of images, and identifying animals initially captured at awkward body angles. This initial trial of a basic camera system has successfully demonstrated that the *ScanEagle* UAV has great potential as a tool for marine mammal aerial surveys.

## Introduction

The conservation and management of many marine mammal species is dependent on monitoring their population status by conducting aerial surveys. For example, in the US, the *Marine Mammal Protection Act* (*MMPA*) *of 1972* requires an annual stock assessment of all marine mammal species in US waters. Many of these stock assessments, and the consequential management actions to conserve marine mammals, are based on minimum population estimates from aerial surveys (e.g., aerial surveys were conducted for many species reported in Waring et al [1]). Similarly, the status assessments of cetaceans by the International Whaling Commission and the IUCN often rely on aerial survey data [2,3,4,5]. The datasets produced from aerial surveys provide basic knowledge of the ecological requirements of species such as manatees (*Trichechus manatus latirostris*) [6], North Atlantic right whales (*Eubalaena glacialis*) [7], humpback whales (*Megaptera novaeangliae*) [8], harbour porpoises (*Phocoena phocoena*) [9], and Risso’s dolphins (*Grampus griseus*) [10]. In addition, aerial surveys can be used to assess the effectiveness of marine mammal sanctuaries (e.g., [11,12,13]), and have been essential for setting catch limits for subsistent hunting [14]. 

In Australia, dugong (*Dugong dugon*) populations have been regularly surveyed over the last 20 to 30 years throughout large parts of their range (e.g., [15,16,17]). Aerial surveys are an effective survey method for this species because the method allows coverage of their extensive range [18]. The overhead perspective of aerial surveys also maximises the detection probability of dugongs considering they spend very little time at the water surface [19]. It is government policy in Queensland to “obtain up-to-date knowledge of dugong populations through continuing aerial surveys at appropriate intervals throughout Queensland coastal waters” [20]. Survey datasets from Queensland, the Torres Strait and the Northern Territory have been used to develop dugong density models to predict critical habitat areas and conduct risk assessments of human impacts [17,21,22]. In Western Australia, the *Shark Bay Marine Reserves Management Plan 1996-2006* (not yet superseded) requires that dugong distribution surveys are conducted throughout the bay (which is 13,000 km^2^) at a minimum of 5-year intervals [23]. In addition, mining in northern Australia has increased rapidly since 2000 [24], resulting in numerous large-scale coastal developments (ports and processing facilities) proposed and approved throughout the dugongs’ range. Therefore, the Environmental Impact Assessments for most of these developments are required to include aerial surveys for dugongs [e.g., 25,26,27]. 

Field and analysis techniques for marine mammal surveys have continually been improved to provide robust abundance estimates [e.g. 28,29,30,31,32]. They are generally flown in a small aircraft at a set altitude and speed along transect-style flight paths designed to minimise biases in sampling. Usually, two to four observers call or record sightings in real time. The surveys can either use (a) strip transect sampling, where all animals are counted within a marked strip width on either side of the aircraft and all are considered equally detectable [e.g. 18], or (b) line transect (distance) sampling, where observers record all sightings, and the density of the animals is calculated based on the probability of seeing an animal according to its distance from the trackline [see 28,33]. For the latter, observers record angles and bearings to each sighting so that the distances from the trackline can be calculated. For both techniques, the GPS locations of the sightings are generally calculated according to the aircraft’s GPS location at the time the sighting was called. 

Recent developments in the technical capacity and civilian use of Unmanned Aerial Vehicles (UAVs, defined as vehicles flying without a human pilot on board) have led to some investigations into the potential use of these systems for aerial surveys of marine mammals [34,35,36,37,38,39]. UAVs operating under autopilot and mounted with GPS and imaging systems have the potential to replace traditional manned aerial surveys and provide an improved method for monitoring marine mammal populations. The standard methods employed for surveys using manned aircraft have a number of limitations, which may be overcome by using UAVs:

1. Human risk – Manned aerial surveys pose a risk to the observers with at least five aircraft crashes having killed 11 marine mammal researchers during aerial surveys [40,41,42,43,44]. The UAV will eliminate this risk to researchers.
2. Costs – There are large costs involved in chartering aircraft, hiring and training observers, and paying for accommodation. Aerial surveys are usually limited to Beaufort sea states of 3 or less [e.g. 45], and therefore the costs include keeping the aircraft and observers on standby waiting for the appropriate weather conditions. As UAVs continue to be developed for civilian use, we hope that the cost of aerial surveys can be reduced through cheaper operating costs, fewer personnel, flying longer hours per day (i.e., there is no need for resting observers and UAVs can fly longer before refueling), and the potential for surveying in a wider range of weather conditions.
3. Missed sightings and/or misidentification of animals – Manned aerial surveys depend on (usually four) experienced observers who are capable of correctly identifying marine mammals to species level [46,47]. This requires significant training. Furthermore, observer bias (proportion of animals visible to the observer but missed) needs to be accounted for in abundance estimates [45]. If UAV imaging systems provide a permanent visual record of sightings of high enough resolution, they should increase the accuracy of detection and identification of species.
4. Low resolution of location data – The location of each animal group sighted during manned surveys has error associated with time lag between observers seeing animals and then calling/marking their location, as well as measurement errors inherent in determining the location of the animals that are some distance from the aircraft (trackline). UAV imaging systems will provide an immediate snapshot of the sighting and an accurate GPS location of the image. There is also the potential to take this further and use the detailed flight logs recorded by the UAV system to obtain the GPS location of each animal within the image.
5. Ability to survey isolated or otherwise inaccessible habitat areas – Some marine mammal species occur in areas that are inaccessible or too dangerous for manned surveys because of their distance from the nearest airstrip or from shore. UAVs do not require an airstrip and can be operated from a ship so may overcome these limitations depending on their range and endurance.

Considering the high demand for dugong aerial surveys in Australia, and the potential for UAVs to eliminate risk to human safety and improve the data collected from these surveys, we conducted a trial in Shark Bay, Western Australia, with the aim of establishing methods for UAV dugong surveys.

The specific objectives of this trial were:

1. Determine the effectiveness of a UAV with a customised imaging system for detecting and identifying dugongs.
2. Test the capabilities of the UAV system for surveying dugongs in a range of environmental conditions.
3. Determine the ideal resolution: area coverage ratio according to altitude (given our imaging system parameters).

Either of the two sampling techniques outlined above (strip and line transect sampling) could conceivably be applied to UAV surveys, but for this research we have focused on the strip sampling technique because this is the standard method for surveying dugongs [18] and is the simplest approach. This was the first trial in Australia to determine the capabilities of UAVs for fauna surveys. Although the focus for this trial was on dugongs, we also note that we were able to identify a range of other species in the images. 

## Methods

### Ethics statement

This research was conducted under authorisation by The Murdoch University Animal Ethics Committee (permit R2365/10) and Department of Environment and Conservation, Western Australia, permits CE002918 and SF007592.

### Study site

The trial was conducted in Shark Bay, which is situated midway along the coast of Western Australia (25°30’S, 113°30’E). The bay is 13,000 km^2^ in area and divided into two embayments separated by the Peron Peninsula. Shark Bay is afforded a high level of protection as both a Marine Park and a World Heritage Area (WHA). One of the values for which the Bay was nominated as a WHA is its large expanses of seagrass meadows (4,000 km^2^) and diversity of seagrass species (12 species) [48]. The area experiences mean annual temperatures of 17-27°C and limited rainfall (mean 224 mm per year). Five full surveys of the bay have produced dugong population estimates of approximately 10,000 dugongs [16,49,50,51, Hodgson, unpublished data]. Both loggerhead (*Caretta caretta*) and green (*Chelonia mydas*) turtles occur throughout the bay [52]. There is also a large population of bottlenose dolphins (*Tursiops aduncas*) [50] and a humpback dolphin (*Sousa chinesis*) population of unknown size [53].

### Unmanned aerial vehicle and imaging system (payload)

Insitu Inc., a subsidiary of The Boeing Company, are one of the largest UAV developers and operators worldwide. Their UAVs have been tested by a number of other researchers investigating the use of UAVs for wildlife monitoring [e.g. 36,37,38,54]. Insitu Pacific Pty Ltd, (Insitu’s Australia-based subsidiary) equipped their *ScanEagle* UAV with a stills camera payload for this trial, and were the operators of the system. The specifications of the *ScanEagle* are provided in Table **1**. The key capabilities of this particular UAV are the relatively large range and long endurance, both of which would allow this system to be used for surveys of the scale necessary for dugongs.

**Table 1:** Specifications for the ScanEagle UAV.

**Dimensions**	Wing Span	10.2 ft	3.11 m
	Length	4.5 ft	1.37 m
**Weights**	Empty Structure Weight	28.8 lb	13.1 kg
	Max Takeoff Weight	44.0 lb	20.0 kg
**Performance**	Max Horizontal Speed	80 knots	41 m/s
	Cruise Speed	48 knots	25 m/s
	Ceiling	19500 ft	5944 m
	Endurance	24+ hours	
**System features**	Propulsion	1.9 hp (1.4 kw), 2-stroke engine	
	Fuel	Gasoline (100 octane unleaded non-oxygenated gas)	
	Navigation	GPS / Inertial	
	Launch	Pneumatic catapult (“Superwedge UAV Launcher”)	
	Recovery	SkyHook wingtip capture (“Skyhook”)	

The imaging system payload for this trial contained a Nikon^®^ D90 12 megapixel digital SLR camera, together with a fixed video camera in the nose. Imagery from the latter camera was viewed in real-time from the Ground Control Station, providing improved situational awareness. The SLR camera was mounted within the airframe pointing directly downwards using a number of shock-absorbing mounts to reduce vibrations. Each image capture was tagged in real-time with GPS information from a dedicated receiver. Image capture was controlled (including start, stop and capture rate) via the Ground Control Station and the capture rate could be scheduled to achieve a prescribed proportion of overlap ensuring complete coverage along the transect lines flown. All images were stored on the camera’s memory card and downloaded post flight. A standard 50 mm lens was used throughout the trial. A polarising filter was fitted to the lens for all flights, and the direction of the polarisation was kept constant. A yellow filter was tested during one flight to determine whether this would lessen the effect of sun glitter (refer to section Image analysis).

**Figure 1:**
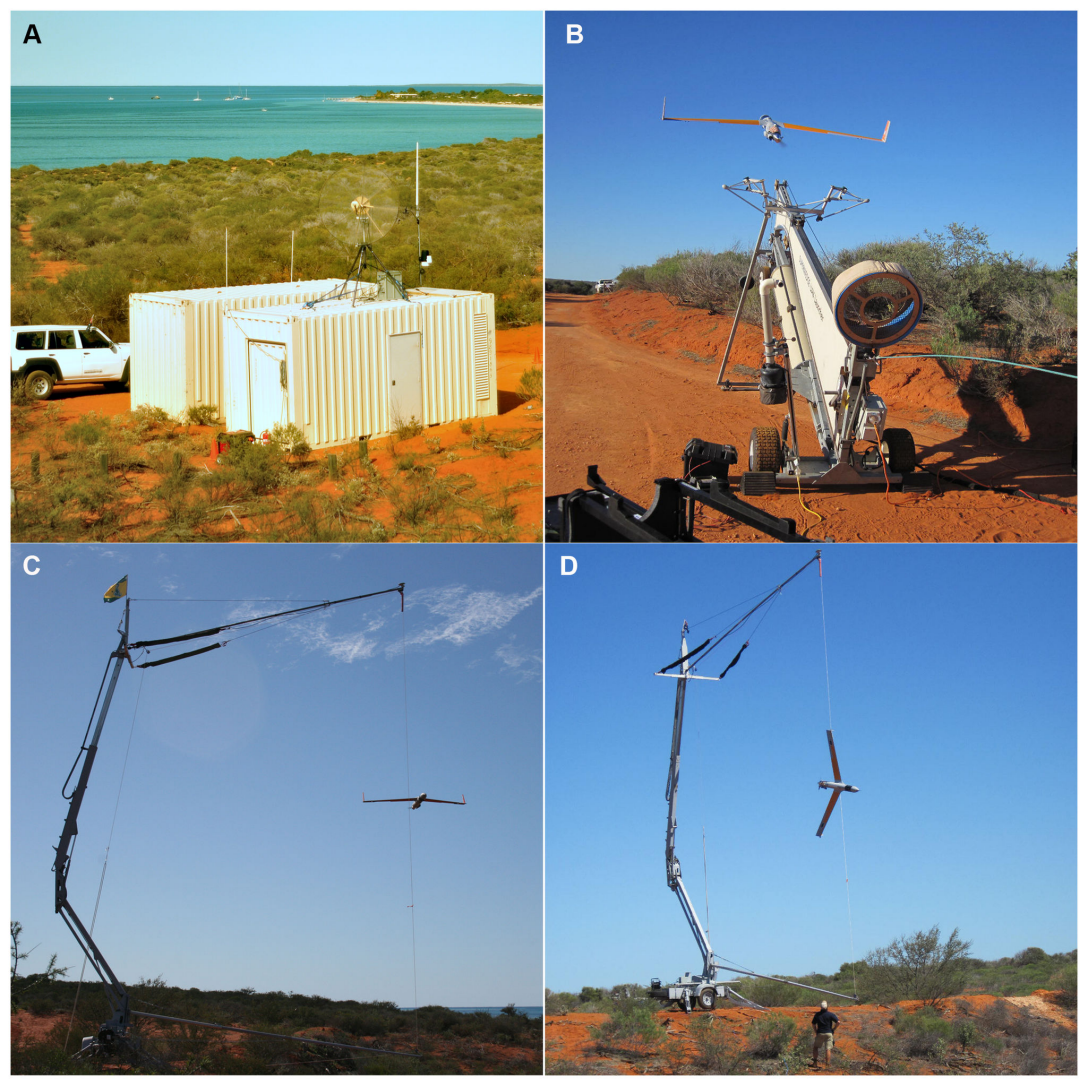
Ground Control Station and launch and retrieval equipment. Photos of (A) the Ground Control Station and storage (inside shipping containers), (B) the Superwedge UAV Launcher, and (C,D) the Skyhook capturing the UAV, set up in Shark Bay Western Australia.

**Figure 2:**
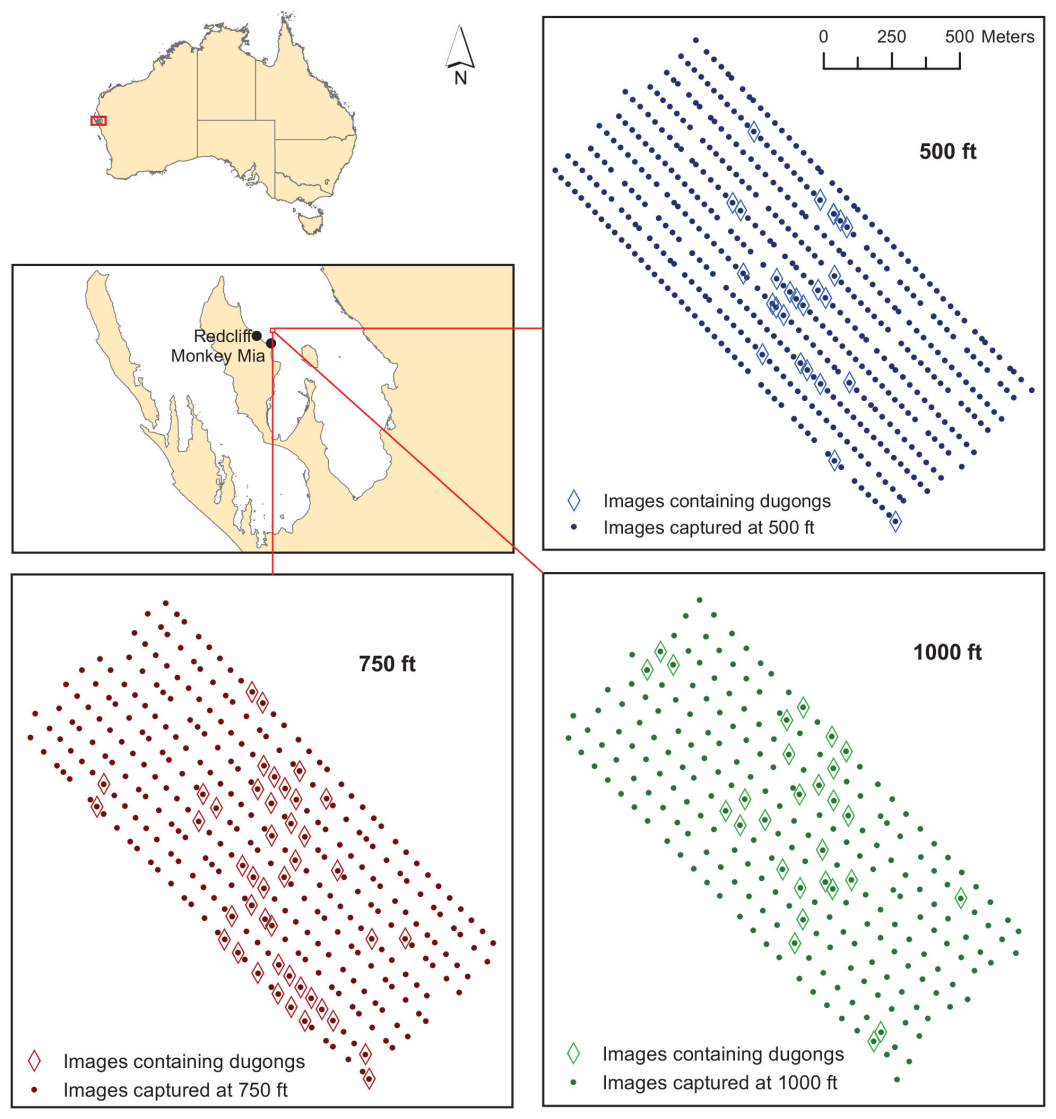
Location of trial and an example of images captured during one flight. Map showing the location of our trials at Shark Bay (the Ground Control station was situated at Redcliff), and as an example, the locations of all images captured from all three surveys during Flight 3. Images containing dugong sightings are highlighted

The UAV Ground Control Station, Superwedge UAV Launcher and Skyhook retrieval system (Figure **1**) were set up at Redcliff, north of Monkey Mia, on the Peron Peninsula, Shark Bay (Figure **2**). This location was chosen because it was accessible by road and allowed the UAV to be kept within line-of-sight as it transited to the survey location (refer to section Range limitations). 

### Range limitations

Our permit from the Australian Civil Aviation Safety Authority (CASA) restricted us to flying the *ScanEagle* only within visual line of sight for this trial. We assigned airspace controllers (ACs) to keep watch on the *ScanEagle* and other aircraft in the area. To extend our flight range, we positioned an AC on land and a second AC on the scout boat, with the duty being passed from one to the other as the *ScanEagle* was flown from the Ground Control Station to the survey area. This ‘visual line of sight’ restriction has been lifted for subsequent UAV trials.

### Flight plan

We programmed the UAV to fly a series of parallel line transects over seagrass banks known to be frequented by dugong herds on a daily basis. Our small ‘survey’ consisted of 10 transects, each 1.8 km in length. The 10 transects were spaced at 72 m intervals, which was the width of view of the water surface within the images when the *ScanEagle* was flown at 500 ft. The width of view at each altitude was the effective transect strip width. The image capture rate was set to achieve 10% overlap in all but one survey, in which this was increased to a simple rate of one image per second. For all surveys, the combination of image capture rate, transect spacing, lens size and altitude provided complete coverage of a 1.296 km^2^ survey area.

We flew the UAV on seven missions (flights), and during each flight we aimed to conduct three surveys, increasing the altitude from 500, to 750 and then 1000 ft (see Figure **2** for an example). We did not alter the spacing of the transects and therefore overlap of the images between transects increased as the altitude increased (Table **2**). It took approximately 1 min to complete each transect and an average of 23 min to complete all 10 transects.

**Table 2:** Details of coverage by images at the three altitudes flown during trial flights.

**Altitude (ft)**	**Field of view (m) ^a^**		**Area of overlap**	
	**Length**	**Width**	**Along transect**	**Between transects**
500	48	72	10%	0 (edge to edge)
750	72	108	10%	25%
1000	96	144	10%	50%

^a^ This is the area of the sea surface visible within each image, where length is along the transect, and width is perpendicular to the transect. Width is equivalent to transect strip width.

The purpose of the trial was to test the capabilities of the imaging system rather than conduct an unbiased survey. Therefore, for each survey we wanted to ensure that the number of images containing dugongs could be maximised. To achieve this goal, the exact location of the survey area, although always on the same seagrass bank, was determined immediately prior to each trial flight according to real time boat-based observations of the dugongs. While the *ScanEagle* was being prepared and launched, a boat was driven to the seagrass bank, a dugong herd was located, the boat was positioned next to the herd, and the boat’s GPS position radioed back to the Ground Control Station. The flight plan (i.e., the mapped locations of the 10 transects) was then altered so that the boat’s GPS position was in the centre of the survey area. Altering the flight plan in this way only took a matter of seconds.

### Image analysis

Post flight, one experienced aerial survey observer (lead author) manually reviewed all images captured whilst transects were being flown. This review consisted of searching for identifiable animals (i.e., including fauna other than dugongs) and scoring the environmental conditions in each image. The Raw images were viewed in ViewNX 2™ (Ver 2.0.1, Nikon^®^, 2010). Sighting data recorded for each image included:

- Species, or where not possible, general taxa.
- Number of individuals of given species / taxa.
- Number of calves.
- Number classified (subjectively) as ‘certain’ and number of ‘uncertain’ individuals; uncertain sightings were either clearly fauna but of unclear taxa, or a ‘dugong shape’ that could not confidently be distinguished within the image.
- Number of double counts (i.e., the same individual animal occurring within the 10% overlap of successive images).

Calves were distinguished because it is important to monitor the proportion of calves in a population when assessing population status. During manned surveys, dugong calves are generally discernible due to their small relative size and their close proximity to their mother. We wanted to ensure that calves are similarly discernible in UAV surveys.

Individual animals resighted (double counted) in successive images along each transect could be identified and were subtracted from the count of individuals for that image. For surveys flown at 500 ft, it was assumed there was no overlap of images between transects because the width of area covered in each image was the same as the distance between the transects. However, at 750 and 1000 ft, there was overlap of images between transects (Table **2**). To account for this, the edges of the images were truncated so that the width of view (transect strip width) was the same for each altitude. All sightings within the truncated edges of the images were discarded, and all results presented herein, include only those sighted within the standard transect strip width of 72 m.

All images containing dugong sightings were then re-checked by the same reviewer to ensure dugong counts and associated sighting data were accurate. 

Three environmental variables were also scored for each image: sea state, turbidity and sun glitter. Sea state was scored for each image according to the Beaufort sea state scale. Turbidity (which incorporated a measure of depth) was subjectively scored for each image captured according to the following categories:

1. Shallow with the bottom clearly visible
2. Shallow with the bottom visible but obscured by turbidity
3. Deep with the bottom not visible, but clear water
4. Deep with the bottom not visible and turbid water

Sun glitter is the sparkling reflection of sun on the water. Each point of light is a specula reflection of the sun, called a sun glint, and glitter is a combination of many sun glints reflecting off wavelets [55]. For our purposes, we distinguish glitter from glare, which is the reflection of diffuse light such as skylight and clouds [56]. The proportion of the image affected by sun glitter is dependent on the position of the sun, the image sensor’s angular field of view, the shape and orientation of the image, and sea surface conditions (wave slope) [56]. We scored sun glitter for each image captured as a subjective estimate of the percentage area of the image affected (0%, <25%, 25-50% and >50%).

### Effects of altitude and environmental conditions

#### Altitude and certainty of sightings

The effect of altitude on the certainty of identifying dugongs was tested within a mixed-effect generalized linear model (implemented with R package *lme4* [57]), where each sample was a sighting of an individual dugong that was either certain or uncertain; ‘flight’ was treated as a random effect; we applied a logit-link and we assumed a Binomial distribution (i.e., logistic regression). Even though clumping of dugongs is known to occur [e.g., 58], the mean number of dugongs per image was less than two; so, for the purposes of this analysis, individual dugongs were treated as an independent sample. A corresponding confidence interval was estimated using a MCMC Bayesian approach (R package *MCMCglmm* [59]). 

#### *Dugong sighting* rates

In order to determine the effects of four covariates—altitude, sun glitter, turbidity and Beaufort sea state—on the dugong sighting rate, we made the following assumptions:

1. The number of dugongs available to be photographed during the survey is equal relative to each of the covariates. (We acknowledge that turbidity, which incorporates a course measure of water depth, is likely correlated with dugong distribution, however in the absence of data to adequately model this relationship, we are assuming turbidity does not influence dugong distribution.)
2. The number of dugongs available to be photographed during a single survey flight remains constant (i.e., throughout that flight, but can vary between flights).
3. Groups of dugongs are distributed randomly throughout the survey area, and there are no systematic trends in the values of the nominated environmental covariates throughout the survey area.

We then tallied the total number of dugongs sighted in each image (the sample unit), which was accompanied by the associated four covariates. Exploratory analysis found no significant co-linearity between these covariates, and so these were treated as independent during model selection.

We fit a generalized linear mixed-effects model (GLMM) to the number of dugongs detected in each of the images for analysis of the relationship between the ability to see dugongs and our covariates. The response variable—the number of dugongs per image—is considered to be Tweedie distributed in order to account for dugongs forming groups [60]. The R package *cplm* [61] was used to fit the Tweedie GLMMs. 

To account for temporal autocorrelation in the number of dugongs in the survey area at any one time, each flight was treated as a random effect; to account for spatial autocorrelation within each flight, each transect was also treated as a random effect, which was nested within flight. Where there were multiple altitudes flown within each flight, the same set of transects were re-used; this is reflected in the modeling by treating each transect as a replicate within a flight. Each random effect was fitted as an intercept only (i.e., we assumed no interaction between the random effect and any of the fixed effects). We assumed compound symmetry in correlations within each level of the random effects, i.e., we considered each image to be similarly correlated with every other image in that level.

The covariates altitude, sun glitter, turbidity and Beaufort sea state were treated as fixed effects. Altitude (500, 750 and 1000 ft) and Beaufort sea state (0-5) entered the models as continuous values. Sun glitter estimates of 0, <25%, 25-50% and >50% entered the models as an ordinal variable 1, 2, 3 and 4. In testing for the effect of turbidity, this variable was specified in two ways to capture both the traditional way in which it is described (i.e., the 1-4 classification outlined above) and a construct designed to describe the interaction between depth and water clarity. For the water depth × clarity specification, the turbidity variable was split into two separate variables, requiring three parameter estimates. These were: a two-level factor that described depth, either shallow (base level of factor) or deep; a two-level factor that described how clear the water was, either clear (base level of factor) or murky; and an interaction term linking the depth and water clarity variables back to the original 1-4 classification. A constraint was applied during model-selection to ensure that depth, clarity and their interaction remained together in the model when testing that specification of turbidity. 

Because the data are counts, we used a logarithmic link function, and we used an offset term to account for the area covered by images (note, the area per image increases with increasing altitudes, as shown in Results). 

We used a backwards selection process to choose the model of best fit for the fixed-effects component. Given the data were non-Normal and overdispersed, a *t*-test on the last variable or interaction term to enter the model was deemed an appropriate tool to decide upon significance (at the 0.05 level) of model terms [62]. We tested all interaction terms (i.e., two, three and four way). For testing any interactions between a covariate(s) and turbidity, the single turbidity variable is used (i.e., instead of the turbidity variable which is split into three components). 

## Results

### Flight details

We conducted seven flights between 16 and 21 September 2010. Details of each flight are provided in Table **3**, including time of day, sun glitter and Beaufort sea state conditions. During two trial flights the camera did not capture a complete set of images. This issue was investigated in the laboratory and for subsequent trials, the LCD screen of the SLR camera is now visible in real time in the Ground Control Station, so we can confirm that images are being captured. During a single flight on 20 September, there was opportunity to repeat the survey five rather than three times. During that flight we conducted a survey at each of the three altitudes, then repeated the survey at 500 and 1000 ft. From herein we refer to these latter two surveys as a separate flight (Flight 6). Throughout the seven flights and all surveys therein, we captured a total of 6243 images.

**Table 3:** Details of all surveys including sun glitter and sea state conditions.

**Flight**	**Date**	**Survey start time**	**Altitude (ft)**	**No. images captured**	**Number of images in sun glitter category**				**Beaufort sea state**
					**0%**	**<25%**	**25-50%**	**>50%**	
1	16/09/2010	11:31	500	476	35	416	25	0	1	
		12:00	750	220	30	178	12	0	1	
2	18/09/2010	8:24	500	702	0	648	51	3	5	
		8:56	750	316	0	138	178	0	4	
		9:49	1000	235	0	44	191	0	4-5	
3	18/09/2010	12:43	500	494	0	0	20	474	3	
		13:15	750	316	0	0	10	306	3	
		13:56	1000	234	0	1	117	116	2	
4 ^a^	19/09/2010	14:24	750	222	0	4	106	112	2	
		14:50	1000	236	0	74	107	55	2-3	
5	20/09/2010	14:27	500	475	0	407	30	38	2-3	
		14:52	750	314	0	305	9	0	2-3	
		15:24	1000	231	0	231	0	0	2-3	
6	20/09/2010	15:51	500	484	4	480	0	0	2-3	
		16:17	1000	240	24	216	0	0	2-3	
7	21/09/2010	12:24	500	494	0	10	98	386	0	
		12:48	750	318	0	24	96	198	0	
		13:17	1000	236	3	130	81	22	0	

^a^ This was the only flight where the yellow filter was used on the SLR camera lens.

### UAV capabilities

The *ScanEagle* was tested in a range of wind conditions; according to data transmitted from the *ScanEagle*, which we noted at the beginning of each survey, the mean wind speed was 13 knots (range 6-26 knots). The *ScanEagle* was capable of maintaining a parallel line flight pattern throughout this range of wind conditions and the locations of images captured during the three surveys conducted during Flight 3 (wind speed of 17 knots for all three surveys) are provided in Figure **2**. The altitude and rotation (roll, pitch and yaw) of the *ScanEagle* were all recorded continuously at the Ground Control Station during each flight. These data can be used to determine the exact coverage of each image to account for slight variations in altitude or orientation of the UAV (see Discussion).

### Animals sighted

It was possible to identify a range of fauna within the images, including dugongs, dolphins, turtles, sharks, rays, seasnakes, fish schools and birds on the water surface. We note that dolphins and turtles could be identified to species level in many cases. However, the research reported here is focused on using dugongs as a case study so further analyses of other animal sighting data were beyond the scope this work.

**Figure 3:**
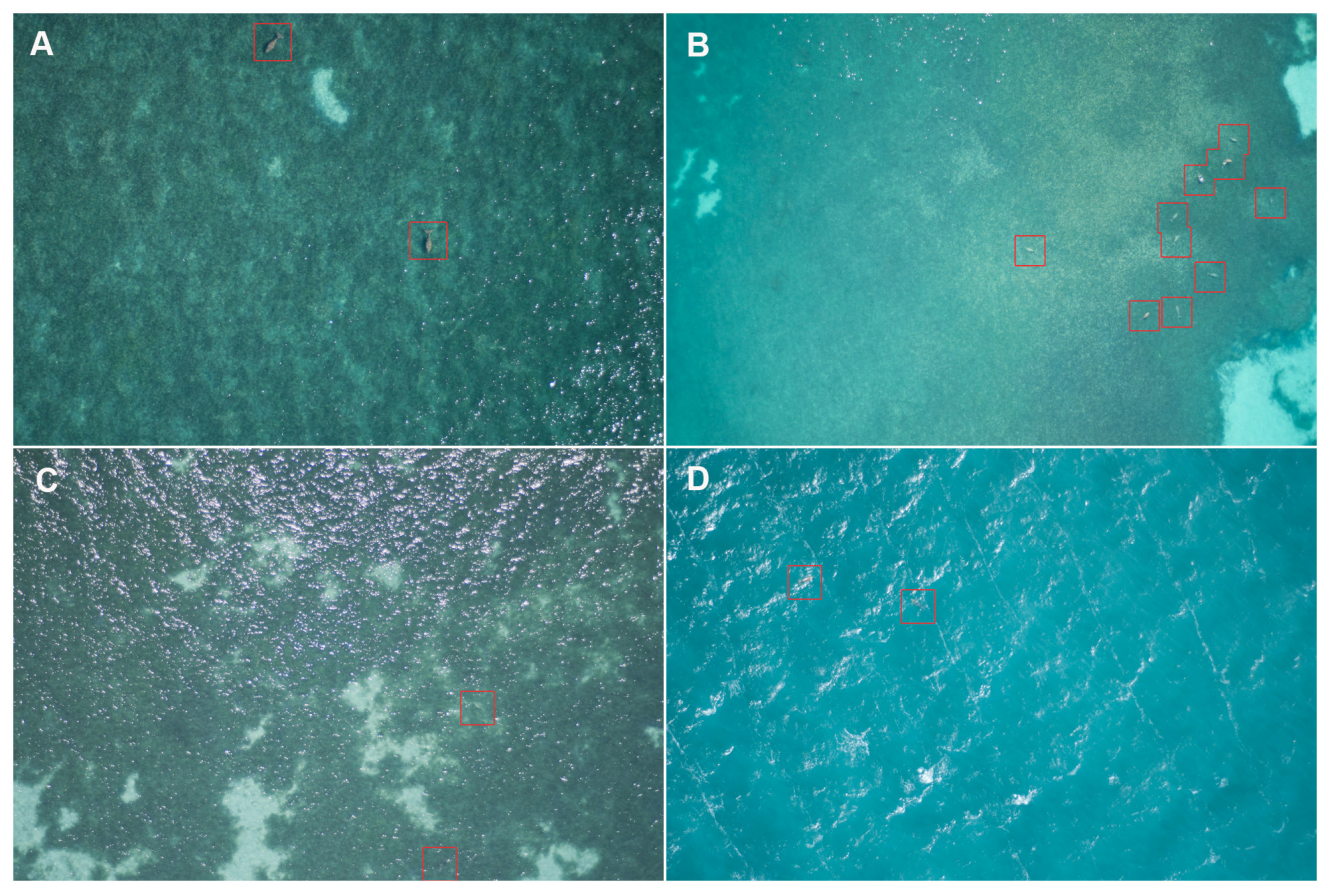
Images with dugong sightings in various conditions and at various altitudes. Example images containing dugong sightings (outlined in red), where (A) was captured at 500 ft, (B) was captured at 1000 ft, (C) is an example of where a dugong visible at the bottom of this image was not visible under the sun glitter in the successive image (750 ft), and (D) was captured during the worst wind conditions (750 ft).

Of all images captured along predefined transect lines, across all surveys, a total of 626 images contained sightings of dugongs. The total count, after eliminating all double counts from overlap of images along the transect line, was 1036 dugong sightings. Of these, 974 dugong sightings were identified with certainty, including 110 calves. Example images containing dugong sightings at each altitude are shown in Figure **3**.

### Altitude and certainty of sightings

The expected proportion of dugongs identified as “certain” (as opposed to “possible but uncertain/unclear”) was 0.95 (95% CI = 0.90, 0.98). This included all sightings, whether at the surface, near the surface or on the seafloor. These proportions did not differ significantly among the three altitudes (χ^2^ test statistic of ^≈^ 0; *p-value* = 0.5). Therefore, dugongs could be identified with the same degree of certainty in images captured at survey altitudes of 500, 750 and 1000 ft.

### Effects of environmental conditions

#### Sun glitter

**Figure 4:**
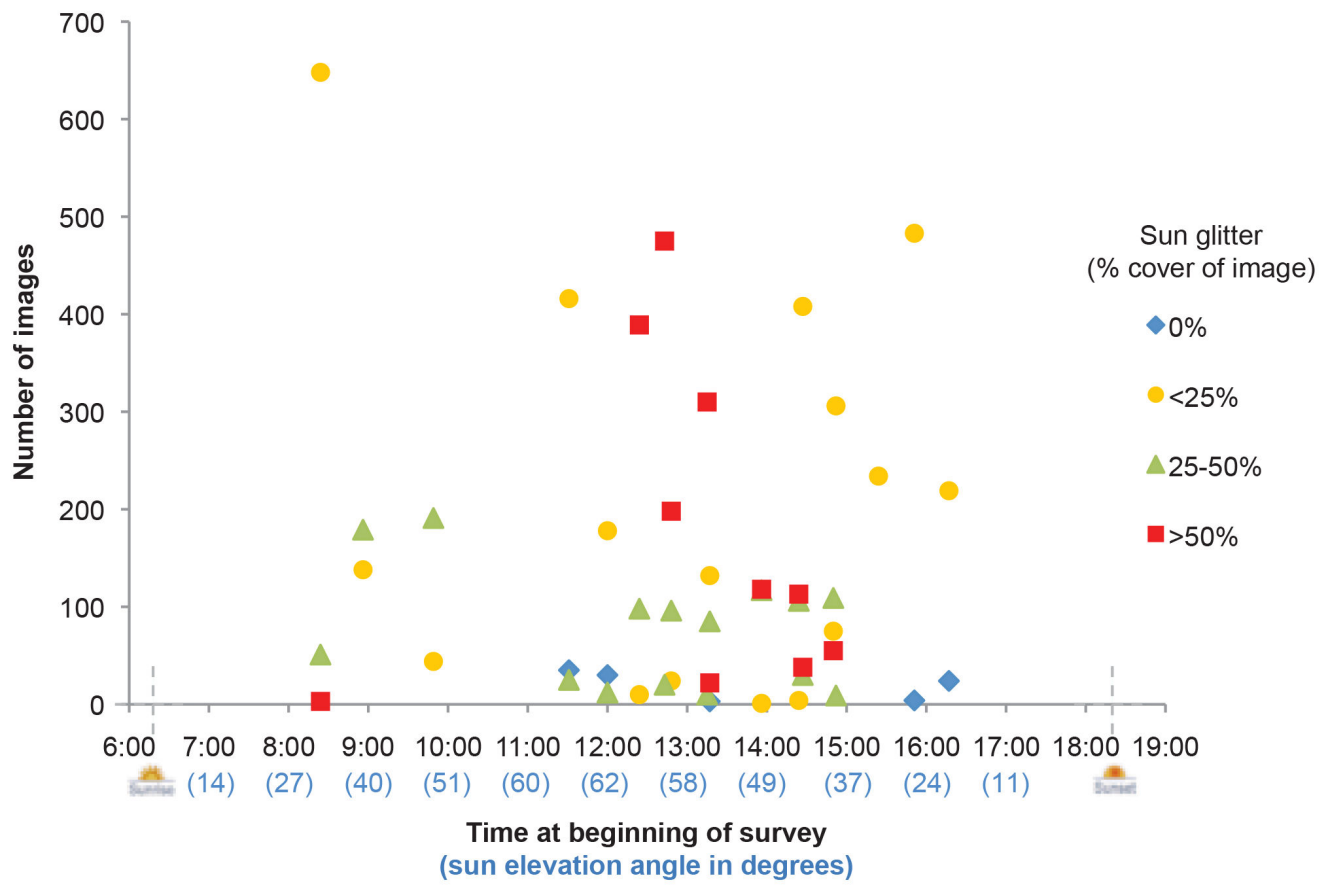
Images affected by sun glitter according to time of day. Number of images within each sun glitter category (a subjective estimate of the percentage area of the image affected) according to the time at the beginning of the surveys, and the corresponding sun elevation angle (calculated using Solar Angle Calculate freeware [67] for 19 September).

Sun glitter was worst during the early afternoon, as shown in Figure **4** where the majority of images in the highest glitter category (> 50% of the image affected) were captured between 12:30 and 3 pm local time. We experienced the highest winds during Flight 2 (18 September), and the resulting high sea state (and thus high wave slope) produced refraction of sun glitter onto the images during the early morning. 

When reviewing the images, the overlap between successive images helped overcome sun glitter issues (see Figure **3C**). It also provided more opportunity to identify animals with certainty. Individuals in one image had often moved before the next image was taken, often meaning the animal was at a better body angle or position in the water column for identification. In some cases there was a difference in clarity (focus) between the two images so that the animal was clearer in one image than the other.

The yellow filter did not appear to reduce the effect of sun glitter, with over half the images captured during the flight on 19 September having 50% or more of the image affected. The polarising filter would only have reduced glare in those images captured at the right angle relative to the sun (see Discussion). There was no obviously discernible effect of the polarising filter on any of the images captured.

#### *Dugong sighting* rate

The GLMM that best accounted for the effects of altitude, sun glitter, turbidity and Beaufort sea state on the dugong sighting rate included turbidity as the only covariate (this was with turbidity specified both as an integer, ordinal value (i.e., 1-4) and split into a ‘depth’ and ‘water clarity’ variable, with an associated interaction, as described above). Therefore, turbidity variables (both specifications) were the only ones found to significantly affect the dugong sighting rate within the images (Table **4**, but only results of water depth × clarity specification for turbidity are reported). Although the random effects of flight and transect were not found to explain a large amount of the variance in the number of dugongs observed in each image, they were retained in the model.

**Table 4:** Parameter estimates from best GLMM to describe number of dugongs per image, as a function of the selected environmental covariate (turbidity; modelled as a function of depth, water clarity and an interaction between these variables).

	**Name**	**Estimate**	**Standard error**	***t*-value**	**Within-random effects correlation**
**Fixed effects**	Intercept	-9.4191	0.2310	-40.77	
	Turbidity: depth	-3.5806	0.2544	-14.08	
	Turbidity: clearness	-1.2481	0.1273	-9.81	
	Turbidity: interaction	4.1117	0.4645	8.85	
**Random effects**	Flight	0.39777			0.091
	Transect	0.27494			0.064
**Residual**		3.97985			

**Figure 5:**
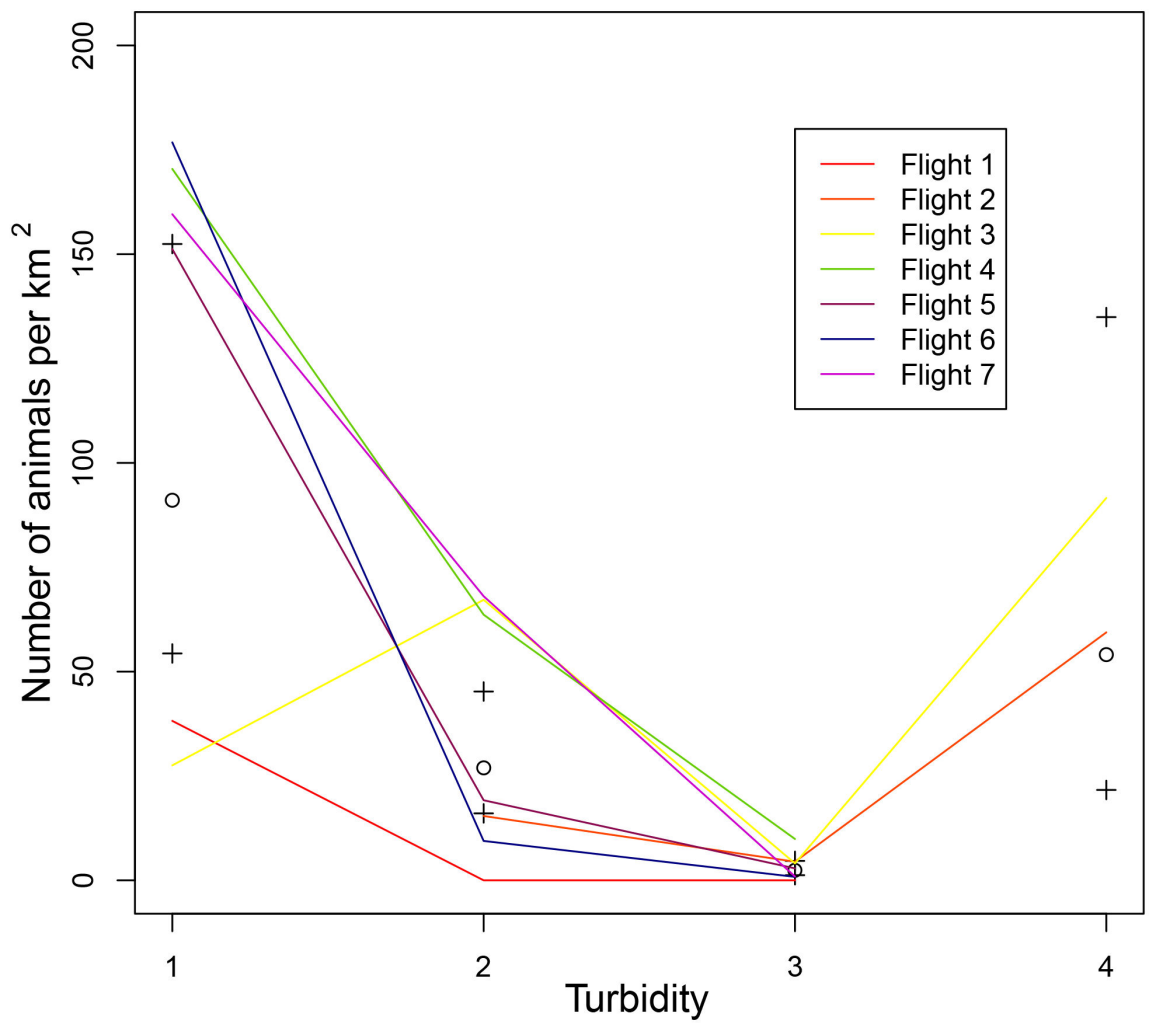
Dugong sightings according to turbidity levels. Sighting rates of dugongs for each UAV flight (adjusted to dugongs per 1.0 km^2^ for ease of comparison) according to the four nominated turbidity levels (1 = shallow with the bottom clearly visible, 2 = shallow with the bottom visible but obscured by turbidity, 3 = deep with the bottom not visible, but clear water, and 4 = deep with the bottom not visible and turbid water); model estimates derived from fixed effects of GLMM and using depth × water clarity specification of turbidity. A comparison of model fit is shown where ○ is the model estimate and + indicates the upper and lower 95% confidence interval on the model estimate.

From Table **4** it follows that—with the assumption that the number of dugongs available to be photographed remained constant across all flight altitudes and environmental covariates tested—in deep water (where the sea floor is not visible because of the depth), the rate of detection of a dugong is 0.03 (where 0.03=exp(-3.5806)) times that for dugongs in shallow waters, keeping water clarity constant. Similarly, in unclear water the rate of detection for a dugong is expected to be about 0.3 (i.e., exp(-1.2481)) times that in clear water, keeping water depth constant. However, there is a significant interaction between depth and water clarity, so when we compare shallow clear water to deep unclear water the system is counter-intuitively better at detecting dugongs in the latter. This may be explained by the lack of sampling in this turbidity category (see Figure **5**; only Flights 2 and 3 sampled in the deep and unclear category for turbidity).

## Discussion

### Image resolution: identifying dugongs and coverage

This initial trial of a basic payload system (digital SLR camera) has successfully demonstrated that the *ScanEagle* UAV has great potential as a tool for marine mammal aerial surveys. A number of animal groups could be readily identified in the images, including dugongs, dolphins, turtles, rays, sharks, seasnakes and birds. Dugongs could be confidently identified and distinguished from other animals such as dolphins or sharks in most cases (expected proportion was 0.95 (95% CI = 0.90, 0.98)). 

Neither our dugong sighting rate, nor our ability to identify dugongs with certainty, were affected by the altitude at which the survey was flown. The *ScanEagle* was flown at three different altitudes: 500, 750 and 1000 ft. Therefore, considering the highest altitude provides the widest transect strip width, the appropriate altitude for a dugong survey (relative to our imaging system parameters) would be a minimum of 1000 ft. This combination of imaging system and altitude provides a strip width at the sea surface of 144 m and equates to approximately 3 cm per pixel. If assuming an average length of a dugong to be 2.5 m, and our standard image clarity applies, our trial suggests that 83 pixels per dugong length is sufficient resolution to identify this species. As we did not conduct flights at altitudes higher than 1000 ft we are not able to comment on whether a lower resolution would be sufficient, or whether a higher altitude would affect image clarity as a result of the amplified effects of any movement of the camera. 

When conducting flights at 1000 ft the transect strip width we achieved (144 m) is narrower than is used during manned surveys, where usually, observers record sightings from 200 m strips on both sides on the aircraft (i.e., the total strip width is 400 m [18]). This means that the *ScanEagle* would need to fly 2.8 times as many transects to achieve the same area coverage that a standard manned survey could achieve. This limitation could be addressed by using a camera that captures images at a higher resolution so that we could use a wider lens or fly higher, or we could use multiple cameras. These options will be assessed in future trials.

### Environmental conditions

Turbidity was expected to influence sighting rates because this variable affects the proportion of time dugongs are available to be sighted, i.e., Pollock et al [29] show that in clear shallow water the probability that dugongs can be seen from the air is 1, however in deep murky water the probability of a dugong being visible from the air is < 0.5. Although our results showed that turbidity influenced sighting rates, the direction of influence was not as expected—the estimate of the sighting rate for dugongs in deep murky waters was higher than those for either deep clear or shallow murky waters. We suggest this is likely a result of the bias in our data because there were so few images in deep murky waters relative to clearer and shallower waters. Our study site typically had clear water. Thus, in order to resolve this potentially spurious result, more trials are needed at a different site with a wider range of water turbidity levels.

We were also expecting that sea state might influence dugong sighting rates. Sea state is known to affect the ability of human observers to sight dugongs during aerial surveys [29]. Dugongs are harder to detect during high sea state conditions presumably because (a) the observers’ eye is drawn to the appearance and movement of white-caps, (b) the white-caps create a masking effect, and (c) turbidity tends to increase. The first two effects are largely removed when capturing still images – it is easier for the observer to look past white-caps that are stationary (see Figure **3D** for an example). We did not detect a high degree of co-linearity between turbidity and sea state, and although turbidity affected sightings rates, sea state did not. This result perhaps suggests that of the three effects of sea state conditions we have outlined, the first (distraction caused by the movement of whitecaps) has the greatest impact on observers detecting dugongs in real time. Our results also suggest that UAV systems may not be limited by sea state conditions in the same manner as manned surveys and that perhaps UAV systems could survey dugongs during higher wind speeds than traditional surveys. This would be a significant advantage, as the cost and logistical constraints of having to conduct manned surveys only during Beaufort sea state of ≤ 3, and thus having charter planes and survey teams waiting on the ground for appropriate conditions, can prohibit surveys, particularly during windy seasons. 

Sun glitter was visible in a high proportion of images, particularly during the early afternoon, but did not affect our dugong sighting rates. During manned aerial surveys, sun glitter also causes problems for observers and survey flights are typically scheduled for early morning and late afternoon to avoid intense midday glitter. In our UAV surveys, however, it appeared that overlap between successive images (along the transect line) overcame the problem of dugongs being masked by sun glitter. This strategy is used in vertical aerial photography for shallow water benthic habitat mapping, where it is suggested that 60% overlap between images compensates for sun glitter [63]. The overlap between our images, although only 10%, still appeared to provide enough information to interpret areas affected by glitter. However, greater overlap would ensure compensation against high levels of sun glitter, would help in identifying animals obscured by white-caps, and may allow stereoscopic analysis of the images. The latter suggestion needs further investigation.

The disadvantage of having large amounts of overlap between images is the large number of images captured and therefore the large amount of memory storage needed, in our case, on board the *ScanEagle*. Although data storage is becoming progressively cheaper and more compact, sun glitter remains a potential issue if surveys are conducted during particular times of the day. Sun glitter can be avoided by calculating the appropriate time of day to survey according to the angle of the sun (zenith angle), wind speeds (which determine wave tilt and therefore reflection of sun glint) and image sensor field of view [56]. Attempting to avoid sun glitter would dramatically limit the flying time available. However, Mount’s [56] equations could instead be used to predict the expected degree of sun glitter on the images and thus determine the proportion of image overlap needed to account for this. Sun glitter can also be minimised by having a small angular field of view (achieved by have a small sensor size and/or long lens), and, if possible, by orientating the short edge of the image towards the sun [56]. 

Using a polarising filter can also reduce reflections of light off the water (both glare and glitter). We recognised that one needs to orient a polarising lens in an appropriate direction relative to the sun in order to filter the reflected light. However, we oriented the lens in a constant direction to determine whether the filter would have an effect in at least some images. We did not detect any obvious reduction in glare or glitter in any particular sets of images. The greatest benefit from the polarising filter occurs when both the sun elevation angle and the camera angle are at 37 degrees from the surface of the water (Brewster’s angle [64]). When conducting vertical photography, there is little to no effect of using a polarising filter, unless using a wide angle lens. The filter does, however, have the undesirable effect of reducing exposure by 1-2 stops [65]. Therefore, for future surveys, where the camera is pointed vertically, we would not recommend using a polarising filter.

### Comparison of the UAV with manned surveys

In our introduction we listed a number of limitations of manned surveys that we suggest UAV surveys may overcome. The first – eliminating human risk – is clearly achieved, because no observers were needed in light aircraft. The second – reducing costs – is difficult to quantify as the commercial company we used sets rates according to the specific requirements of each job and therefore it is not appropriate to quote exact costs. However, during this early stage in the development of UAV methods, Insitu Pacific aim to make their costs at least competitive with manned surveys. Considering this, our trial suggested that UAVs could reduce survey costs by reducing the time needed to complete a survey through (a) the flexibility in acceptable wind conditions, (b) the option to overcome masking of sightings due to sun glitter by overlapping successive images, and (c) the ability to fly longer hours within a day without having to refuel or rest observers. A shorter survey time frame would provide a more robust ‘snapshot’ of animal distribution and abundance across the entire survey area because there is less opportunity for significant animal movement.

The advantage of having a permanent record of each sighting is that images can be re-checked to ensure sighting data are accurate, and strict criteria can be applied for eliminating uncertainties. Reviewing images in consultation with other experts can also increase the accuracy of species identification. Future assessments of UAV sighting data could include an effort to assess observer bias by having multiple experienced people review a subset of images. This idea is conceptually the same as having multiple observers in a manned aircraft; the aim is to maximise detection probability and calculate observer bias using a capture-recapture approach [29,45]. In terms of accuracy of species identification, we again note that the image reviewer was able to confidently distinguish dugongs from other species for an expected proportion of 0.95 (95% CI = 0.90, 0.98) of all dugongs identified across all surveys, including all sightings whether at the surface, near the surface or on the seafloor. 

Manual review of the images is time consuming, however, and the efficacy of UAV surveys will depend on the development of image analysis algorithms to automate the detection of animals (or possible sightings) within the images. There are currently no published algorithms that can detect marine fauna in aerial images, although a number of research groups are attempting to develop software for this purpose. This problem is particularly challenging relative to other areas of image processing automation because (a) marine fauna often occur in images that are made complex by the sea floor, white caps and sun glitter, and (b) the size, colour and shape of the animal can change according to its position in the water column. Although we have suggested here that UAV surveys may not be limited by the same sea state and sun glitter conditions that limit manned surveys, our results apply only to images that have been manually reviewed. The development of software that can automate the processing of images from UAVs could allow us to quantify detection error of the algorithm as a proxy for observer bias, and eliminate the error caused by human fatigue (from reviewing images or observing during manned surveys). However, whether such an algorithm could overcome the challenges of high sea state and high glare conditions remains untested.

The next step in establishing the efficacy of replacing manned aerial surveys with UAVs is to determine whether the proportion of dugongs available to be sighted from the air at any one moment is equivalent for both methods. A great deal of work has been conducted to estimate availability corrections for manned dugong surveys [29,45]. It is now important to determine whether the same proportion of dugongs can be seen in aerial images (i.e., are available) as can be seen by human observers in an aircraft. To this end, a direct comparison between the two techniques might be appropriate.

Acquiring the GPS location of every image provides greater accuracy in location data than for manned surveys where observers are calling sightings. In strip transect sampling, observers typically do not call the position of the animal relative to the trajectory of the aircraft (i.e., how far forward or aft of abeam they sighted the animal). This sampling method is generally used for animals that surface briefly or occur in high numbers, meaning there is no time to measure the position of each sighting – it is either inside the strip or not. Therefore, if there is a 5 second window of opportunity to see an animal as the aircraft passes any one location, the animal could be located anywhere within a 250 m length of space. Even using distance sampling methods, where horizontal and vertical angles are measured, a few seconds delay in taking the measurements will affect the accuracy of the calculated location. During our UAV surveys, detailed data are recorded about the *ScanEagle’s* position, altitude and rotation (pitch, roll and yaw) during each flight. Using this data, the capability of obtaining a coordinate for the corners of each image, and each animal sighted within an image, has been developed and is currently being tested within the software *Vadar* (www.cyclops-tracker.com).

During this trial we were not able to test the ability of the *ScanEagle* UAV to survey in otherwise inaccessible habitat areas. Our flight range was limited to within visual line of sight – a restriction imposed by CASA to prevent conflict with other pilots who were not issuing radio calls and had not read the NOTAM (Notice to Airmen) issued by Insitu Pacific about our operations. This restriction has since been lifted for subsequent surveys conducted within controlled airspace. Assuming permission to fly outside of visual line of sight, the theoretical operational limits of the UAV are (a) range, (b) endurance and (c) transport of the launcher, retrieval system and Ground Control Station. The range of the *ScanEagle* is up to 100 km (depending on topography), with the ability to leapfrog control to relay stations and extend the range. The endurance is up to 24 hours (depending on the weight of the payload) so surveys could conceivably be conducted throughout the day without landing, assuming there is sufficient memory on board for recording images. Transport of the system is a consideration when working in remote areas. However, the *ScanEagle* system used during our trial can also be operated from a ship [e.g., 38], offering further options for accessing remote areas, as well as surveying pelagic species.

Additional benefits of the UAV are the reductions in fuel consumption and potential noise disturbance. The *ScanEagle* UAV consumes 330 ml of fuel per hour, while the standard aircraft used for dugong surveys in Australia, the Partenavia P68, consumes approximately 75-90 L per hour. Even considering the power required to run the Ground Control Station, this provides a significant reduction in the carbon emissions produced by aerial surveys. The *ScanEagle* also produces much less noise than this standard manned aircraft; at maximum throttle the *ScanEagle* noise levels are 85-90 db(A) at 6 m from the aircraft, while the Partenavia P68 produces approximately 80 db(A) at an average distance of 9 km from an airport runway [66].When flown at 1000 ft, it is unlikely that noise from the *ScanEagle* would be audible to marine fauna underwater.

There is currently greater capacity to fly UAVs in civilian airspace in Australia than in some other countries such as the US, because of the regulations set by aviation safety authorities. However, the development of this technique provides alternatives in countries where UAVs are permitted and where accessibility to suitable aircraft and runways are limited. For such applications, a more mobile UAV system may be needed that can be easily transported internationally, but as stated above, the range and endurance of the UAV are also important considerations and can influence the size and transportability of the overall system. Camera systems can also be added to manned-aircraft surveys to gain some of the benefits listed. The trial and analysis presented in this paper, and many of the questions raised, are also relevant to that context.

## Conclusions

This UAV trial showed that dugongs could be readily detected within images captured using the *ScanEagle* with a digital SLR imaging system. There are a number of potential advantages to using the UAV to conduct surveys rather than a manned aircraft. An unexpected outcome was that the dugong sighting rate using the imaging system was not affected by sea state, while high sea states are known to decrease sighting rates during manned surveys. Therefore UAV surveys could potentially be conducted in a wider range of wind conditions than manned surveys. The two key factors inhibiting the application of UAV surveys are (a) the field of view achieved within an image, which limits coverage within each transect, and (b) the time taken to analyse images manually in lieu of an image analysis algorithm that automates the detection of sightings. 

## References

[1] Waring GT, Josephson E, Maze-Foley K, Rosel P, editors (2011) US Atlantic and Gulf of Mexico Marine Mammal Stock Assessments - 2010. NOAA Tech Memo NMFS NE . p. 219. p. 598. Available: National Marine Fisheries Service , 166 Water Street, Woods Hole, MA 02543-1026, http://www.nefsc.noaa.gov/nefsc/publications/.

[2] Hammond PS, Berggren P, Benke H, Borchers DL, Collet A et al. (2002) Abundance of harbour porpoise and other cetaceans in the North Sea and adjacent waters. J Appl Ecol 39: 361-376. doi:10.1046/j.1365-2664.2002.00713.x.

[3] Heide-Jørgensen MP, Borchers DL, Witting L, Laidre KL, Simon MJ et al. (2008) Estimates of large whale abundance in West Greenland waters from an aerial survey in 2005. J Cetacean Res Manag 10: 119-129.

[4] Paxton CGM, Hedley SL, Bannister JL, Group IV Humpback (2005) whales: their status from aerial and land-based surveys off Western Australia. J Cetacean Res Manag (In press).

[5] Lowry L, O'Corry-Crowe G, Goodman D (2006) Delphinapterus leucas (Cook Inlet subpopulation). IUCN 2011. IUCN Red List of Threatened Species. Version 2011.2. Available: http://www.iucnredlist.org . Accessed 2012 Jan 11

[6] Craig BA, Reynolds JE (2004) Determination of manatee population trends along the Atlantic coast of Florida using a Bayesian approach with temperature-adjusted aerial survey data. Mar Mamm Sci 20: 386-400. doi:10.1111/j.1748-7692.2004.tb01168.x.

[7] Keller CA, Ward-Geiger LI, Brooks WB, Slay CK, Taylor CR et al. (2006) North Atlantic right whale distribution in relation to sea-surface temperature in the southeastern United States calving grounds. Mar Mamm Sci 22: 426-445. doi:10.1111/j.1748-7692.2006.00033.x.

[8] Bannister JL, Hedley SL (2001) Southern Hemisphere Group IV humpback whales: their status from recent aerial surveys 47. Memiors of the Queensland Museum pp. 587-598.

[9] Sonntag RP, Benke H, Hiby AR, Lick R, Adelung D (1999) Identification of the first harbour porpoise (*Phocoena* *phocoena*) calving ground in the North Sea. J Sea Res 41: 225-232. doi:10.1016/S1385-1101(98)00050-1.

[10] Baumgartner MF (1997) The distribution of Risso's dolphin (*Grampus* *griseus*) with respect to the physiography of the northern Gulf of Mexico. Mar Mamm Sci 13: 614-638. doi:10.1111/j.1748-7692.1997.tb00087.x.

[11] Marsh H (2000) Evaluating management initiatives aimed at reducing the mortality of dugongs in gill and mesh nets in the Great Barrier Reef World Heritage Area. Mar Mamm Sci 16: 684-694. doi:10.1111/j.1748-7692.2000.tb00965.x.

[12] Slooten E, Rayment W, Dawson S (2006) Offshore distribution of Hector's dolphins at Banks Peninsula, New Zealand: is the Banks Peninsula Marine Mammal sanctuary large enough? N Z J Mar Freshw Res 40: 333-343. doi:10.1080/00288330.2006.9517425.

[13] Panigada S, Lauriano G, Burt L, Pierantonio N, Donovan G (2011) Monitoring winter and summer abundance of cetaceans in the Pelagos Sanctuary (Northwestern Mediterranean Sea) through aerial surveys. PLOS ONE 6: e22878. doi:10.1371/journal.pone.0022878. PubMed: 21829544. 21829544 PMC3146501

[14] Heide-Jorgensen MP, Laidre KL, Burt ML, Borchers DL, Marques TA et al. (2010) Abundance of narwhals (Monodon monoceros) on the hunting grounds in Greenland. J Mammal 91: 1135-1151. doi:10.1644/09-MAMM-A-198.1.

[15] Marsh H, Lawler IR, Kwan D, Delean S, Pollock K et al. (2004) Aerial surveys and the potential biological removal techniques indicate that the Torres Strait dugong fishery is unsustainable. Anim Conserv 7: 435-443. doi:10.1017/S1367943004001635.

[16] Holley DK, Lawler IR, Gales NJ (2006) Summer survey of dugong distribution and abundance in Shark Bay reveals additional key habitat area. Wildl Res 33: 243-250. doi:10.1071/WR05031.

[17] Grech A, Sheppard J, Marsh H (2011) Informing species conservation at multiple scales using data collected for marine mammal stock assessments. PLOS ONE 6: e17993. doi:10.1371/journal.pone.0017993. PubMed: 21464933. 21464933 PMC3065465

[18] Marsh H, Sinclair DF (1989) An experimental evaluation of dugong and sea turtle aerial survey techniques. Aust Wildl Res 16: 639-650. doi:10.1071/WR9890639.

[19] Hodgson AJ (2004) Dugong behaviour and responses to human influences [PhD]. Townsville: James Cook University. 295 pp.

[20] EPA (2010) Operational policy: Conservation and management of dugongs in Queensland. Review Date: August 2010. Brisbane, Queensland: Enivronmental Protection Agency p. 8.

[21] Grech A, Marsh H (2007) Prioritising areas for dugong conservation in a marine protected area using a spatially explicit population model. Appl GIS 3: 1-14.

[22] Grech A, Marsh H (2008) Rapid assessment of risks to a mobile marine mammal in an ecosystem-scale marine protected area. Conserv Biol 22: 711-720. doi:10.1111/j.1523-1739.2008.00923.x. PubMed: 18410398. 18410398

[23] CALM (1996) Shark Bay Marine Reserves. Management Plan: 1996-2006, Management Plan No 34. Perth, Western Australia: Department of Conservation and Land Management for The National Parks and Nature Conservation Authority . 114 p

[24] Connolly E, Orsmond D (2011) The mining industry: from bust to boom. Research Discussion Paper. RDP 2011-08 Sydney: Reserve Bank of Australia.

[25] Chevron (2012) Wheatstone Project: Dugong Research Plan. Perth: Chevron Australia Pty Ltd.

[26] RPS (2010) Browse Liquefied Natural Gas Precinct Strategic Assessment Report (Draft for Public Review) December 2010. Appendix C-9: Nearshore Regional Survey Dugong Report. Subiaco, Western Australia: RPS 75 p.

[27] GHD (2009) Marine megafauna baseline and impact assessment. Report for Western Basin Dredging and Disposal Project, Gladstone Ports Corporation. Brisbane: October: GHD

[28] Buckland ST, Anderson DR, Burnham KP, Laake JL, Borchers DL et al. (2001) Introduction to Distance Sampling: Estimating abundance of biological populations. Oxford: Oxford University Press.

[29] Pollock K, Marsh H, Lawler IR, Aldredge MW (2006) Estimating animal abundance in heterogeneous environments: an application to aerial surveys for dugongs. J Wildl Manag 70: 255-262. doi:10.2193/0022-541X(2006)70[255:EAAIHE]2.0.CO;2.

[30] Fewster RM, Southwell C, Borchers DL, Buckland ST, Pople AR (2008) The influence of animal mobility on the assumption of uniform distances in aerial line-transect surveys. Wildl Res 35: 275-288. doi:10.1071/WR07077.

[31] Borchers D, Marques T, Gunnlaugsson T, Jupp P (2010) Estimating Distance Sampling Detection Functions When Distances Are Measured With Errors. J Agric, Biol, Environ Statist 15: 346-361. doi:10.1007/s13253-010-0021-y.

[32] Buckland ST, Laake JL, Borchers DL (2010) Double-Observer Line Transect Methods: Levels of Independence. Biometrics 66: 169-177. doi:10.1111/j.1541-0420.2009.01239.x. PubMed: 19432793. 19432793

[33] Buckland ST, Anderson DR, Burnham KP, Laake JL, Borchers DL et al., editors (2004) Advanced Distance Sampling. Oxford: Oxford University Press.

[34] Jones GP IV, Pearlstine LG, Percival HF (2006) An assessment of small unmanned aerial vehicles for wildlife research. Wildl Soc Bull 34: 750-758. doi:10.2193/0091-7648(2006)34[750:AAOSUA]2.0.CO;2.

[35] Koski WR, Allen T, Ireland D, Buck G, Smith PR et al. (2007) Evaluation of an unmanned airborne system for monitoring marine mammals. Anchorage, Alaska: Paper SC/59/E1 presented to the IWC Scientific Committee, 7-18 May 2007. 16 p.

[36] Buck GB, Ireland D, Koski WR, Sliwa D, Allen T et al. (2007) Stategies to improve UAS performance for marine mammal detection. Anchorage, Alaska: Paper SC/59/E2 presented to the IWC Scientific Committee, 7-18 May 2007. 15 p.

[37] Koski WR, Abgrall P, Yazvenko SB (2009) A review and inventory of unmanned aerial systems for detection and monitoring of key biological resources and physical parameters affecting marine life during offshore exporation and production activities. Madeira, Portugal: Paper SC/61/E9 presented to the IWC Scientific Committee, 31 May - 12 June 2009. 12 p.

[38] Moreland EE, Cameron M, Boveng PL, Angliss RP (18-22 1 2010). urveys Seals Bering Sea Pack ICE Using Unmanned Aircraft Systems; 2010

[39] Martin J, Edwards HH, Burgess MA, Percival HF, Fagan DE et al. (2012) Estimating distribution of hidden objects with drones: from tennis balls to manatees. PLOS ONE 7: e38882. doi:10.1371/journal.pone.0038882. PubMed: 22761712. 22761712 PMC3382610

[40] Stone G (1988) Memories. Mar Mamm Sci 4: 276. doi:10.1111/j.1748-7692.1988.tb00209.x.

[41] O'Shea TJ, Ackerman BB, Percical HF, editors (1995) Population Biology of the Florida Manatee. Washington DC: US Department of the Interior, National Biological Service, Information and Technology Report 1. 289 p

[42] Cosens SE, Maiers L, Chambers A, Cleator H, Dunn B (2000) H Stuart Innes (1953-2000). Arctic 53: 330-331.

[43] Wells M (2003) Wildlife trust mourns loss of right whale survey team. Mar Mamm Soc Newsletter 11: 8.

[44] ASFC (2008) AFSC Quarterly Research Reports for July - August - September 2008. Washington DC (available online http://www.afsc.noaa.gov : Alaska Fisheries Science Center.

[45] Marsh H, Sinclair DF (1989) Correcting for visibility bias in strip transect aerial surveys of aquatic fauna. J Wildl Manag 53: 1017-1024. doi:10.2307/3809604.

[46] Laake JL, Calambokidis J, Osmek SD, Rugh DJ (1997) Probability of detecting harbor porpoise from aerial surveys: Estimating g. J Wildl ManagVolumes 0: 61: 63-75

[47] Hobbs RC, Waite JM (2010) Abundance of harbor porpoise (Phocoena phocoena) in three Alaskan regions, corrected for observer errors due to perception bias and species misidentification, and corrected for animals submerged from view. Fish Bull Seattle 108: 251-267.

[48] Walker DI (1989) Seagrass in Shark Bay - the foundations of an ecosystem. In: Larkum AWD McComb AJ Shepherd SA Biology of Seagrass: A treatise on the biology of seagrasses with special reference to the Australian region. Amsterdam: Elsevier pp. 182-210.

[49] Marsh H, Prince RIT, Saalfeld WK, Shepherd R (1994) The distribution and abundance of the dugong in Shark Bay, Western Australia. Wildl Res 21: 149-161. doi:10.1071/WR9940149.

[50] Preen AR, Marsh H, Lawler IR, Prince RIT, Shepherd R (1997) Distribution and abundance of dugongs, turtles, dolphins and other megafauna in Shark Bay, Ningaloo Reef and Exmouth Gulf, Western Australia. Wildl Res 24: 185-208. doi:10.1071/WR95078.

[51] Gales NJ, McCauley RD, Lanyon JM, Holley DK (2004) Change in abundance of dugongs in Shark Bay, Ningaloo and Exmouth Gulf, Western Australia: evidence for large scale migration. Wildl Res 31: 283-290. doi:10.1071/WR02073.

[52] Heithaus MR, Frid A, Wirsing AJ, Bejder L, Dill LM (2005) Biology of sea turtles under risk from tiger sharks at a foraging ground. Mar Ecol Prog S 288: 285-294. doi:10.3354/meps288285.

[53] Parra GJ, Corkeron PJ, Marsh H (2004) The Indo-Pacific humpback dolphin, *Sousa* *Chinensis* (Osbeck, 1765), in Australian waters: a summary of current knowledge. Aquat Mamm 30: 197-206. doi:10.1578/AM.30.1.2004.197.

[54] Koski WR, Allen T, Ireland D, Buck G, Smith PR et al. (2009) Evaluation of an unmanned airborne system for monitoring marine mammals. Aquat Mamm 35: 348-358.

[55] Lynch DK, Livingston W (1995) Color and light in nature. Cambridge: Cambridge University Press. 254 pp.

[56] Mount R (2005) Acquisition of through-water aerial survey images: Surface effects and the prediction of sun glitter and subsurface illumination. Photogrammetric Eng Remote Sensing 71: 1407-1415.

[57] Bates D, Maechler M, Bolker B (2011) lme4: Linear mixed-effects models using S4 classes. R package version 0.999375-42. Available: http://CRAN.R-project.org/package=lme4.

[58] Anderson PK (1982) Studies of dugongs at Shark Bay, Western Australia I. Analysis of population size, composition, dispersion and habitat use on the basis of aerial survey. Aust Wildl Res 9: 69-84. doi:10.1071/WR9820069.

[59] Hadfield JD (2010) MCMC methods for multi-response generalized linear mixed models: The MCMCglmm R Package. J Stat Softw 33: 1-22. PubMed: 20808728.20808728

[60] Williams R, Hedley SL, Branch TA, Bravington MV, Zerbini AN et al. (2011) Chilean blue whales as a case study to illustrate methods to estimate abundance and evaluate conservation status of rare species. Conserv Biol 25: 526-535. doi:10.1111/j.1523-1739.2011.01656.x. PubMed: 21385211. 21385211

[61] Zhang W (2012) Compound Poisson linear models. R Package version 0.6-4. Available: http://cran.r-project.org/web/packages/cplm/.

[62] Bolker BM, Brooks ME, Clark CJ, Geange SW, Poulsen JR et al. (2009) Generalized linear mixed models: a practical guide for ecology and evolution. Trends Ecol Evol 24: 127-135. doi:10.1016/j.tree.2008.10.008. PubMed: 19185386. 19185386

[63] Finkbeiner M, Stevenson B, Seaman R (2001) Guidance for benthic habitat mapping: An aerial photographic approach. Charleston SC: US NOAA Coastal Services Center. Available: http://www.csc.noaa.gov/.

[64] Brewster D (1815) On the laws which regulate the polarisation of light by reflection from transparent bodies. Philos Trans R Soc Lond 105.

[65] Kuzina AM, Ramm NS, Semenchenko IV (1960) Use of polarization light filters in aerial photographic sea surveys. News of Institutes of Higher Learning of the USSR Ministry of Specialized Higher and Intermediate Education: Geodesy and Aerial Photographic Surveys Section Issue No. 6: 83-95

[66] Australia Airservices (2013) Noise and flight path monitoring system Melbourne Quarterly Report ML12Q4 October - December 2012.

[67] Forster. Engineering Services P/L (2012); Angle Calculator Solar. Available: http://aie.org.au/Content/NavigationMenu/Resources/EnergyData/Solar_Angle_Calcula.htm . Accessed 18 January 2012

